# Lower or higher HDL-C levels are associated with cardiovascular events in the general population in rural China

**DOI:** 10.1186/s12944-020-01331-6

**Published:** 2020-06-25

**Authors:** Shasha Yu, Xiaofan Guo, Guang Xiao Li, Hongmei Yang, Liqiang Zheng, Yingxian Sun

**Affiliations:** 1 Department of Cardiology, First Hospital of China Medical University, 155 Nanjing North Street, Heping District, Shenyang, 110001 China; 2 Department of Clinical Epidemiology, Institute of Cardiovascular Diseases, First Hospital of China Medical University, Shenyang, 110001 China; 3 Department of Clinical Epidemiology, Shengjing Hospital of China Medical University, Shenyang, 110004 China

**Keywords:** Cardiovascular events, HDL-C, Cardiovascular mortality, Rural, General, Risk, Lipid, Incidence

## Abstract

**Background:**

The present study aims to estimate whether high-density lipoprotein cholesterol (HDL-C) is correlated with cardiovascular events (CVEs) and cardiovascular mortality (CVM) in a large sample of the general population in rural areas of China.

**Methods:**

Adult participants (*n* = 10,266, age = 53.79 ± 10.49 years; 46.5% men) were enrolled from the Northeast China Rural Cardiovascular Health Study (NCRCHS). Laboratory testing, blood pressure, weight, height, and questionnaires about socioeconomic status were collected.

**Results:**

In all, 585 nonfatal or fatal CVEs and 212 cardiovascular deaths were documented during a 4.66-year follow-up. Compared to the reference groups (HDL-C between 1.5 and 1.99 mmol/L), either lower or higher levels of HDL-C were correlated with an increased incidence of CVEs but not CVM [hazard ratio (HR) _the lowest_ = 1.369, 95% confidence interval, 1.007–1.861; HR _the highest_ = 1.044, 0.509–2.231]. Elevated CVM was seen in the lowest HDL-C category (1.840; 1.121–3.021).

**Conclusions:**

Lower or higher HDL-C was associated with a higher incidence of CVEs but not CVM in the general population of rural China. Perhaps if an appropriate level of HDL-C is maintained, CVEs can be effectively prevented.

## Background

Growing lines of evidence indicate that high-density lipoprotein cholesterol (HDL-C) has a close relationship with all-cause mortality, especially cardiovascular mortality (CVM) [1, 2]. Some research has shown that elevated HDL-C levels are accompanied by a decreased rate of coronary artery diseases and CVM [3]. Conversely, others have reported that extremely high concentrations are also related to higher all-cause mortality risk [4–6]. Similarly, the AIM-HIGH study indicated that HDL-C is a useful marker to predict cardiovascular events (CVEs) [7]. Low HDL-C has been suggested to be responsible for the higher incidence of CVEs. It seems that the higher the HDL-C value is, the more beneficial the effect on the cardiovascular (CV) system. However, many therapies targeting an increase in HDL-C have failed to show consistent clinical benefits [8, 9]. Thus, focus has recently shifted towards evaluating whether only a lower HDL-C concentration is associated with a higher incidence of CVEs.

In the recent past, most studies have intended to estimate whether HDL-C is associated with CVEs or CVM and have enrolled subjects from urban or high-income areas. Data from high-income countries show a markedly decreasing trend in the incidence and mortality of CVEs. However, for middle- and low-income countries or areas, the rates of CVEs and CVM are still increasing dramatically and account for the majority of the burden in these areas [10–12]. In China, rural areas are characterized by significantly lower socioeconomic levels than urban areas. A previous study confirmed that although the risk-factor burden increases with socioeconomic levels, the incidence and fatality rate of CVEs exhibit a reverse pattern. For example, there was a lower prevalence of total CVEs in higher-income areas (7.46%) than in lower-income areas (8.36%) [13]. The researchers determined that, except for income levels, lifestyle, health service level, frequency of effective treatments, and educational status of the subjects were all accountable for this discrepancy. Subjects living in rural areas had relatively lower income and educational status and less medical support. Therefore, the rates of CVEs and mortality were higher among rural subjects. On the other hand, people living in rural Northeast China have unique lifestyles, including being more prone to eat vegetables, having higher physical activity intensity due to farm work and experiencing less stress [14, 15]. All these lifestyle characteristics might somehow alleviate CV risk factors.

The Northeast China Rural Cardiovascular Health Study (NCRCHS) was an epidemiological study conducted to estimate CV risk factors, CVEs and CVM among rural subjects. At baseline, participants had a higher rate of CV risk factors, such as dyslipidaemia [high total cholesterol (TC): 16.4%; low HDL-C: 13.8%; high LDL-C: 7.6%; high triglyceride: 17.3%], hypertension (51.1%), diabetes (15.3%), and hyperuricaemia (10.9%) [14–17]. Due to the increasing trend of CVEs and mortality in rural areas, it is necessary to evaluate the possible correlation between HDL-C and CVEs or CVM to better prevent and control those CV risk factors among rural residents. Furthermore, in China, urban subjects have a significantly higher rate of statin use than rural residents (6.83% vs. 2.5%). As health propagation gradually becomes widespread in rural areas, an increasing number of rural residents who have dyslipidaemia will receive lipid-lowering medication treatment. Estimation of whether these subjects are suitable for receiving lipid-lowering medication should be done before starting medication treatment. If a higher HDL-C level is associated with a higher risk of CVEs or mortality among rural residents, more concern should be paid to these subjects. Hence, it is necessary to evaluate whether lower or higher HDL-C is relevant to CVEs or mortality among rural subjects.

## Methods

### Participants

A community-based prospective study, named NCRCHS, was conducted in rural China [18]. Baseline data were collected from 2012 to 2013. In total, the present study enrolled 11,956 participants (older than 35 years). The China Medical University Ethics Committee approved the present study (Shenyang, China, AF-SDP-07-1, 0–01). Written informed consent was signed by all participants who agreed to participate in the present study. Participants took part in one physical examination, including anthropometric measurements [body weight (BW), waist circumference (WC), height, hip circumference], blood pressure measurements, and blood tests (routine blood and biochemical tests). They completed one questionnaire for the collection of relevant information, such as medical history, family history and socioeconomic factors. The details of the study design have been reported in previous papers, which were all cross-sectional analyses [19–21]. To better evaluate the possible CV risk factors among rural residents, a follow-up study was conducted from 2015 to 2017. In the follow-up study, participants at baseline were invited to attend the follow-up study. In total, 1256 out of 11,956 subjects were excluded due to a lack of contact information. Ultimately, 10,266 participants finished the follow-up visits (median 4.66 follow-up years). All participants signed written informed consent forms. Covariables with complete information from the baseline visit were used in the present analysis. The present study included data from both the baseline study and the follow-up study, which distinguishes this study from previous papers.

### Study variables

Self-reported history of stroke, coronary heart disease (CHD) and chronic heart failure at baseline were recorded and confirmed by medical records. Participants were requested to wear lightweight clothing and take off their shoes when their weight and height were measured. WC was measured as previously described [18]. Obesity was defined using body mass index (BMI) criteria with the cut-off ≥28 kg/m^2^ [BMI equal to weight (kg) divided by height squared (m) ^2^] [22]. Blood pressure was measured automatically following the standard criteria using an electronic sphygmomanometer (HEM-907; Omron, Tokyo, Japan). Systolic blood pressure (SBP) greater than 140 mmHg and/or diastolic blood pressure (DBP) greater than 90 mmHg, with or without medication, were defined as hypertension [23]. After fasting for at least 12 h, participants provided blood samples, which were obtained by trained nurses. Fasting plasma glucose (FPG) and lipid profiles, such as low-density lipoprotein cholesterol (LDL-C), HDL-C, TC and triglycerides, were analysed enzymatically. FPG greater than 7 mmol/L and/or the use of antidiabetic drugs indicated diabetes [24].

Annual income of the family meant the total income of the family in the whole year and was categorized into ≤5000 CNY/year and > 5000 CNY/year (1CNY = 0.161 USD). Physical activity, including occupational and leisure-time physical activity, was described in detail previously [18].

### Adjudication of endpoints

In the present analysis, incident CVEs included a composite of new-onset stroke or CHD during the follow-up period. When participants reported a possible diagnosis or death, all the available clinical data, such as medical records, relevant imaging data and death certificates, were collected. The end-point assessment committee was responsible for reviewing and adjusting the data independently.

### Statistical analysis

Mean values and standard deviations were used to describe continuous variables, whereas categorical variables are reported using numbers and percentages. Kaplan-Meier estimates were adopted to compute the cumulative incidence of CVEs for each group, and the log-rank test was used to compare the differences in estimates. Cox proportional hazards models were used to identify the relationship between HDL-C and the risk of CVEs and CVM incidence, and hazard ratios (HRs) together with 95% confidence intervals (CIs) were calculated. Age, sex, smoking (never, ex-smoker, current smoker), physical activity (light, moderate, severe), nutritional status (diet scores), overweight or obesity, hypertension, diabetes, longstanding illness, and TC were adjusted in the model. The curvilinear trend was estimated by adding a squared term for HDL-C. SPSS version 17.0 software was used to perform all the statistical calculations, and *P* less than 0.05 indicated statistical significance.

## Results

The present research included 10,266 participants (53.79 ± 10.49 years; 46.5% men). HDL-C ≥ 2.5 mmol/L was identified in 1.4% of participants, with a higher proportion of men in this group (Table 1). Subjects in the very high HDL groups had higher rates of current smoking and alcohol consumption, lower annual income and higher SBP, DBP, and TC but lower rates of light physical activity and chronic diseases and BMI values.

**Table 1:** Baseline Characteristics of the present study

	High-Density Lipoprotein Cholesterol Category, mmol/L					
Variables	< 1.0	1.0–1.49	1.50–1.99	2.0–2.49	≥2.5	***P*** value
**Age, mean ± SD**	53.11 ± 10.50	53.82 ± 10.47*	53.74 ± 10.50	54.41 ± 10.43*	55.64 ± 10.13*^#†^	0.023
**Men (%)**	57.5	44.4*	43.4*	53.7^#†^	70.8*^#†‡^	< 0.001
**Current smokers (%)**	39.3	33.2*	34.5*	45.8*^#†^	61.1*^#†‡^	< 0.001
**Current drinker (%)**	14.6	18.7*	26.7*^#^	43.2*^#†^	70.1*^#†‡^	< 0.001
**Physical activity**		*	*^#^	*^#†^	*^#^	< 0.001
**Light**	41.5	37.2	33.9	26.5	27.1	
**Moderate**	20.2	19.0	18.1	16.0	17.4	
**Severe**	38.3	43.8	48.0	57.5	55.6	
**Annual income < 5000 CNY/year**	11.4	11.1	12.4	13.9	24.3*^#†‡^	< 0.001
**Chronic diseases (%)**	28.3	24.9*	23.8*	20.9*^#^	19.4*^#^	0.003
**Total Cholesterol, mmol/L**	4.66 ± 0.93	5.19 ± 1.07*	5.41 ± 1.05*^#^	5.70 ± 1.16*^#†^	5.96 ± 1.32*^#†‡^	< 0.001
**Low density Lipoprotein Cholesterol, mmol/L**	2.69 ± 0.73	2.98 ± 0.83*	2.99 ± 0.85*	3.00 ± 0.89*	2.81 ± 0.87^#†‡^	< 0.001
**Systolic blood pressure, mmHg**	141.15 ± 23.71	141.11 ± 23.17	143.15 ± 23.69*^#^	145.48 ± 22.88*^#†^	151.83 ± 25.23*^#†‡^	< 0.001
**Diastolic blood pressure, (mmHg)**	82.25 ± 12.14	81.89 ± 11.66*	81.96 ± 11.67*	82.65 ± 11.79	84.53 ± 12.67^#†^	0.001
**Body mass index, kg/m**^**2**^	26.35 ± 3.71	25.17 ± 3.62*	24.07 ± 3.61*^#^	23.32 ± 3.10*^#†^	22.99 ± 3.23*^#†^	< 0.001

^*^P < 0.05 vs. < 1.0 mmol/L, ^#^*P* < 0.05 vs. 1.0–1.49 mmol/L, ^†^*P* < 0.05 vs. 1.50–1.99 mmol/L, ^‡^*P* < 0.05 vs. 2.0–2.49 mmol/L.

**Fig. 1:**
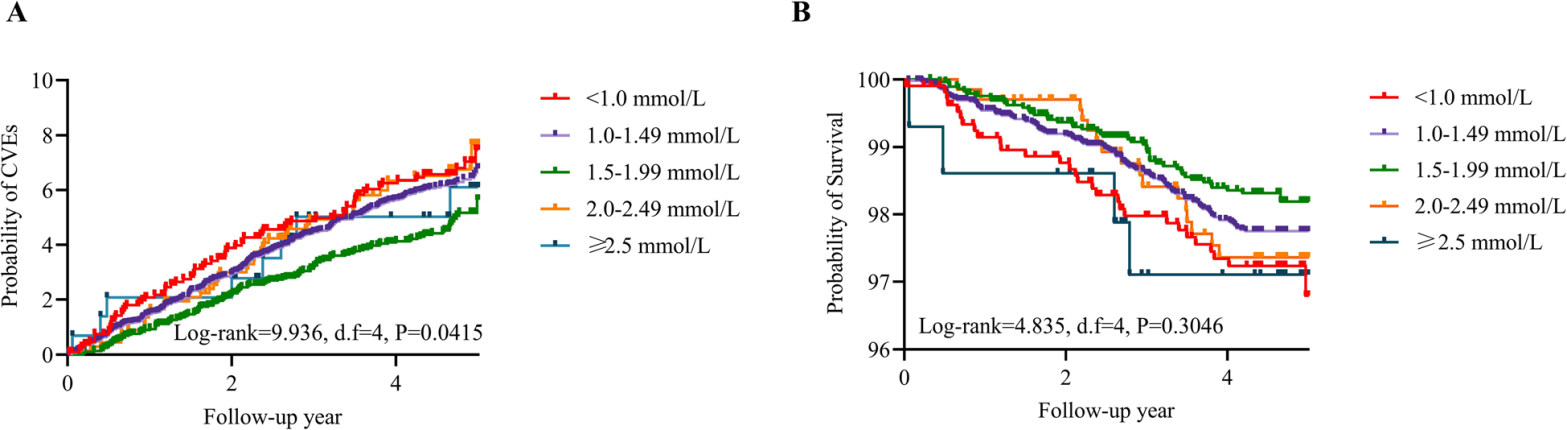
A. Survival and cardiovascular events by different HDL-C levels; B. Survival and cardiovascular mortality by different HDL-C level

There were 585 nonfatal or fatal CVEs and 212 CV deaths during the 4.66-year follow-up. Kaplan-Meier estimates were used to compute the cumulative incidence of CVEs for each group. The data in Fig. 1 show that there was a significant difference in CVE probability (*P* = 0.0416) but no significant difference in CVM (*P* = 0.3046). When further compared with the reference category (1.5–1.99 mmol/L), the risk of CVEs was higher in the lower HDL-C (< 1.0 mmol/L and 1.0–1.49 mmol/L) and higher HDL-C (2.0–2.49 mmol/L) categories after adjusting for possible confounders (Table 2).

**Table 2:** Association Between High-Density Lipoprotein Cholesterol and Cardiovascular Events and Mortality (n = 10,266)

HDL-C Category, mmol/L	n	CVD Events (fatal and nonfatal)	Hazard Ratio (95%CI) CVD events	CVD Deaths	Hazard Ratio (95%CI) CVD Deaths
**< 1.0**	1063	70 (6.59)	1.369(1.007–1.861)	29(2.73)	1.840(1.121–3.021)
**1.0–1.49**	5509	330(5.99)	1.256(1.019–1.548)	116(2.11)	1.317(0.925–1.874)
**1.5–1.99**	2879	132(4.58)	1.000 (Ref)	47(1.63)	1.000 (Ref)
**2.0–2.49**	671	44(6.56)	1.466(1.032–2.081)	16(2.38)	1.508(0.849–2.678)
**≥2.5**	144	9(6.25)	1.044(0.509–2.231)	4(2.78)	1.427(0.509–4.003)
***P*curvilinear trend**			0.038		0.157

Model adjusted for age, sex, smoking (never, ex-smoker, current smoker), physical activity (light, moderate, severe), nutritional status (diet scores), overweight or obesity, hypertension, diabetes, longstanding illness, total cholesterol

In total, 212 deaths were ascribed to cardiovascular disease (CVD) with no evidence of a curvilinear trend. In comparison with the reference groups, an increase in CVM existed only in the lowest HDL-C category (Table 2).

Participants with existing CVD at baseline (16.2% of subjects) who reported a physician diagnosis of CVD were removed, although the results were not changed between HDL-C categories and the incidence of CVD (Table 3). However, the lowest HDL-C category was no longer associated with CVM.

**Table 3:** Association Between High-Density Lipoprotein Cholesterol and Cardiovascular Events and Mortality after removal of participants with existing CVD at baseline (*n* = 9455)

HDL-C Category, mmol/L	n	CVD Events (Fatal and nonfatal)	Hazard Ratio (95%CI) CVD events	CVD Deaths	Hazard Ratio (95%CI) CVD Deaths
**< 1.0**	950	52(5.47)	1.439(1.011–2.049)	19(2.00)	1.759(0.972–3.184)
**1.0–1.49**	5063	269(5.31)	1.377(1.087–1.744)	88(1.74)	1.371(0.916–2.053)
**1.5–1.99**	2674	103(3.85)	1.000 (Ref)	37(1.38)	1.000 (Ref)
**2.0–2.49**	631	37(5.86)	1.509(1.024–2.224)	13(2,06)	1.532(0.808–2.905)
**≥2.5**	137	8(5.84)	1.228(0.595–2.534)	4(2,92)	1.696(0.598–4.808)
***P*curvilinear trend**			0.012		0.115

Model adjusted for age, sex, smoking (never, ex-smoker, current smoker), physical activity (light, moderate, severe), nutritional status (diet scores), overweight or obesity, hypertension, diabetes, longstanding illness, total cholesterol

## Discussion

The present study aimed to estimate whether lower or higher HDL-C levels were correlated with a higher incidence of CVEs and mortality among rural subjects. The results showed that either lower or higher HDL-C was associated with a higher incidence of CVEs, whereas only lower HDL-C increased CVM.

HDL-C plays key roles in the pathways related to the development of atherosclerotic disease, including reverse transportation of cholesterol, oxidation, inflammation, and the maintenance of vascular endothelial function [25]. The measurement of HDL-C is highly utilized by clinicians to predict CV risk. David L Laitinen and colleagues found that a 0.026 mmol/L increase in HDL-C from baseline was related to a 1.3% decreased risk of major CVEs (including CVD-related diagnoses or invasive CV procedures) [26]. Similarly, a cross-sectional analysis revealed a strong, independent, inverse association between HDL-C concentrations and established CVD, even at ranges of HDL-C considered normal [27]. The lack of a U-shaped relationship between HDL-C and CVEs in previous studies might be due to the variation in the criteria used to divide HDL-C categories [1, 24]. The cut-off point shift made the U-shaped relationship known.

Most previous studies focused on the association between HDL-C and CVM [28–30]. Others claimed that HDL-C concentrations were inversely associated with the occurrence of CVEs (CVD-related diagnoses or any of the invasive CV procedures) [26]. The present study reported for the first time that both lower and higher HDL-C can be relevant to increasing the risk of CVEs among rural Chinese individuals. Similarly, a previous study reported that active increases in serum HDL-C concentrations failed to reduce CVD risk [31]. These findings challenged the classic HDL-C hypothesis that higher concentrations of HDL-C are more beneficial for the CV system. One possible reason that the higher HDL-C did not show a beneficial effect on CVEs is that even with a greater amount of HDL-C, the function of HDL-C might be compromised [32]. Cumulative clinical evidence has shifted investigative attention from the HDL-C concentration to its physiological function [33]. HDL exerts many anti-atherogenic properties, such as anti-inflammation, antioxidation, anti-apoptosis, and vasodilating effects. However, among these effects, HDL-C efflux capacity is assumed to be a major anti-atherogenic property of HDL and is used to describe the ability to promote cholesterol efflux from macrophages [34]. HDL-C efflux capacity has been proven to be relevant to carotid intima-media thickness, the incidence of CVD risk and CVM [35, 36]. HDL-C and apoA-1 concentrations have a close relationship with cholesterol efflux capacity. However, this does not necessarily mean that increasing HDL-C will result in higher cholesterol efflux capacity. Therefore, a possible explanation for the association of higher HDL-C with an increased incidence of CVEs might be due to cholesterol efflux dysfunction. Further experiments are needed to clarify this hypothesis.

The results from the present study revealed that in the higher categories of HDL-C, the rates of current smoking and alcohol consumption and the levels of blood pressure and TC were significantly higher than those in the lower categories. This might be one of the reasons for the higher incidence of CVEs in the higher HDL-C groups [37]. One interesting finding is that a previous study revealed a pervasive pattern in which subjects who had relatively lower cholesterol levels had relatively lower incomes, worse lifestyle factors, and less physical activity [1]. However, in the present study, among rural Chinese individuals, participants with higher HDL-C had a higher rate of lower income (24.3% vs. 11.4%), light physical activity (27.1% vs. 41.5%) and unfavourable lipid profiles than those with lower HDL-C. This might also contribute to the observation of the association between lower or higher HDL-C and CVEs among rural Chinese individuals.

### Study strengths and limitations

A strength of the study was that this was the first study to evaluate whether lower or higher HDL-C was correlated with CVEs or mortality among subjects from rural China. The importance of HDL-C evaluation in rural subjects should be further emphasized. More attention should be focused on HDL-C levels during risk management to prevent CVEs and mortality.

The current work has several limitations that must be considered when interpreting the results. First, the recruited subjects came from one province of Northeast China; therefore, the findings cannot be generalized to all subjects across the country. Second, there was some participant loss of contact during the follow-up, which might cause bias in the relationship between HDL-C and CVEs or CVM. Third, the association between HDL-C and the incidence of CVEs and mortality was based on a single blood test, which might result in bias. Fourth, medication status, such as steroid usage, was not collected in detail in the present study, which might cause high HDL-C levels, which might partially affect the relationship between HDL-C categories and CVEs or CVM. Finally, it is well known that fat quality in the diet is correlated with lipid profiles. Therefore, dietary components might affect the association between HDL-C levels and CVEs or CVM. However, in the present study, there was a lack of dietary information resulting in the inability to explain the increased HDL level and the simultaneous increased risk of CVEs.

## Conclusion

The present study confirmed that either too low or too high HDL-C is associated with the incidence of CVEs among the rural Chinese population. In addition, for CVM, only the very low HDL level was related to a higher risk of CVM. This relationship became nonsignificant once subjects with CVD at baseline were excluded. One famous Chinese sentence is “going beyond is just as wrong as not going far enough”. Perhaps keeping HDL-C at a moderate level is the best option for the CV health.

## Data Availability

Enquiries regarding the availability of primary data should be directed to the principal investigator Professor Yingxian Sun (sunyingxiancmu1h@163.com).

## Abbreviations

CVEs: Cardiovascular events
CVM: Cardiovascular mortality
CVD: Cardiovascular disease
BMI: Body mass index
SBP: Systolic blood pressure
DBP: Diastolic blood pressure
FPG: Fasting plasma glucose
TC: Total cholesterol
LDL-C: Low-density lipoprotein cholesterol
HDL-C: High-density lipoprotein cholesterol
TG: Triglyceride
